# Novel Customizable Fracture Fixation Technique vs. Conventional Metal Locking Plate: An Exploratory Comparative Study of Fixation Stability in an Experimental In Vivo Ovine Bilateral Phalangeal Fracture Model

**DOI:** 10.3390/ma18143359

**Published:** 2025-07-17

**Authors:** Thomas Colding-Rasmussen, Nanett Kvist Nikolaisen, Peter Frederik Horstmann, Michael Mørk Petersen, Daniel John Hutchinson, Michael Malkoch, Stine Jacobsen, Christian Nai En Tierp-Wong

**Affiliations:** 1 Department of Orthopaedic Surgery, Rigshospitalet, University Hospital, Blegdamsvej 9, 2100 Copenhagen, Denmark; 2 Department of Orthopaedic Surgery, Hvidovre University Hospital, Kettegaard Alle 36, 2650 Copenhagen, Denmark; 3 Department of Veterinary Clinical Sciences, Faculty of Health and Medical Sciences, University of Copenhagen, Agrovej 8, 2630 Copenhagen, Denmark; 4 Department of Orthopaedic Surgery, Herlev-Gentofte University Hospital, Gentofte Hospitalsvej 1, 2900 Copenhagen, Denmark; 5 Department of Fibre and Polymer Technology, KTH Royal Institute of Technology, Brinellvägen 8, 114 28 Stockholm, Sweden

**Keywords:** fracture fixation, biomechanical stability, in vivo ovine fracture model

## Abstract

A novel composite patch osteosynthesis technique (CPT) has demonstrated promising ex vivo biomechanical performance in small tubular bones. To bridge the gap toward clinical evaluations, this study compared the stability of the CPT to a stainless-steel locking plate (LP) in an experimental in vivo ovine bilateral proximal phalanx fracture model. Eight sheep underwent a midline osteotomy with a 4.5 mm circular unicortical defect in the lateral proximal phalanx of both front limbs, treated with the CPT (n = 8) or the LP (n = 8). A half-limb walking cast, or a custom off-loading hoof shoe, was used for postoperative protection. Implant stability was assessed by post-surgery X-ray evaluations and post-euthanasia (16 weeks) dual-energy X-ray absorptiometry (DXA). At week one, all CPT implants demonstrated mechanical failure, while all LPs remained overall intact. Mean BMD was 0.45 g/cm^2^ for CPT and 0.60 g/cm^2^ for LP in the fracture area (*p* = 0.078), and 0.37 g/cm^2^ vs. 0.41 g/cm^2^ in the distal epiphysis (*p* = 0.016), respectively. In conclusion, the CPT demonstrated indications of inferior stability compared to the LP in this fracture model, which may limit its clinical applicability in weight-bearing or high-load scenarios and in non-compliant patients.

## 1. Introduction

Open reduction internal fixation (ORIF) using prefabricated metal implants is considered a standard approach for managing bone fractures due to their well-established biomechanical characteristics and biocompatibility [1,2]. However, certain fractures, such as osteoporotic, complex multifragmented, and those near vital anatomical structures, may benefit from customized implants to optimize fracture fragment reduction and biomechanical conditions while minimizing tissue damage and inflammation [3,4,5]. A novel in situ customizable composite-based fracture fixation technique, the ‘composite patch technique’ (CPT), has been proposed as an alternative to conventional metal implants [6,7]. This technique employs a prototype thiol-ene-based viscous material that is anchored to the bone using conventional cortical screws, moulded into a desired shape in situ, and cured with a handheld high-energy visible (HEV) light source [6].

The CPT is a novel prototype not yet optimized for a specific fracture type. Its initial introduction in 2021 proposed use in low-load-bearing bones, such as phalanges and metacarpals, due to its demonstrated ability to reduce soft tissue adhesions in an in vivo rat femur model, a key concern in surgical fixation of hand fractures [6,8,9]. Based on this rationale, subsequent ex vivo biomechanical studies focused on small tubular bones, comparing CPT to conventional stainless steel locking plates. In an ex vivo ovine proximal phalanx model, the CPT showed higher bending stiffness in reduced fractures and higher torsional stiffness in non-reduced fractures [7]. Further, in a porcine metacarpal model, stability comparable to corresponding metal locking plates under low to moderate cyclic loading (10–70 N) was demonstrated [6]. Although all previous ex vivo studies of the CPT found that the ultimate mechanical strength of the CPT was lower than the corresponding metal LP, one study demonstrated sufficient functional stability of the CPT in an ex vivo human proximal phalanx fracture model, with the CPT withstanding full finger flexion and grip forces representative of daily activities [10]. Further, an internal load estimation of the proximal phalanx fracture during full finger flexion indicated that the ultimate strength of the CPT exceeded estimated physiological demands more than 100-fold, justifying moving forward towards in vivo animal testing [10].

Therefore, based on previous ex vivo biomechanical studies, the CPT may offer a viable fixation option for selected low-load-bearing fractures such as phalanx and metacarpal fractures. However, the mechanical limitations of the material and its performance under physiological loading conditions remain unknown. Accordingly, to advance toward clinical applications, large-animal in vivo validation and comparison with a conventional LP are needed to confirm the promising results observed in previous studies.

To our knowledge, no established large-animal fracture model exists for small tubular, low-load-bearing bones. We therefore developed a bilateral ovine proximal phalanx fracture model with a specific focus on animal welfare, avoiding restrictive slings and instead applying off-loading coaptation measures using a walking cast and/or a custom-made off-loading hoof shoe. The detailed welfare characteristics of the novel fracture model are presented in a separate manuscript beyond the scope of this paper. This study evaluates and compares the in vivo fixation stability of the CPT to a stainless-steel LP using this novel experimental model. Based on previous biomechanical studies, we hypothesize that the CPT will demonstrate mechanical stability comparable to the LP under in vivo conditions.

## 2. Materials and Methods

### 2.1. Study Overview

This study compared the biomechanical stability of the CPT and LP repair of proximal phalangeal osteotomies in a novel experimental in vivo ovine model. This novel model involved a standardized mid-diaphyseal osteotomy with a circular defect in the proximal phalanx, designed to mimic clinical fracture complexity and enable future evaluation of bone-healing-promoting materials. Off-loading was achieved using a hoof block under the medial (non-operated) digit or a half-limb walking cast, both established strategies in veterinary surgery, which effectively allowed the animals to move freely post-surgery. Fracture healing and implant stability were monitored by intraoperative and postoperative radiographic evaluation and post-surgery DXA scanning of bone mineral density (BMD). A detailed report of the animal welfare outcomes is reported elsewhere; however, all surgical and methodological details relevant to this study are provided here.

Eight sheep were utilized, each undergoing a mid-diaphyseal osteotomy on the lateral proximal phalanx of both front limbs. The osteotomy was treated with the CPT or a 5-hole stainless steel LP (Locking Plate 1.5, straight, Depuy Synthes, Zuchwil, Switzerland), randomly assigned to the left or right limb, allowing each sheep to serve as its control (Table 1).

Fracture fixation stability was evaluated using X-ray imaging intraoperatively and at 1 week post-surgery. The original protocol included weekly radiographic monitoring up to 16 weeks; however, due to early mechanical failure of the CPT constructs, extended radiographic follow-up was not reported for these cases. Dual-energy X-ray absorptiometry (DXA) was performed post-euthanasia at week 16 to assess bone mineral density (BMD) in the fracture area and distal epiphysis.

In the first three weeks post-surgery, the sheep were evaluated by clinical examinations and pain assessments, including lameness evaluations 1–5 times daily by veterinarians, and were evaluated by the animal caretakers two times daily. Welfare was assessed by an established clinical severity score (CSS) [11]. In the subsequent 13 weeks, evaluations occurred several times per day by animal caretakers and 3 or more times weekly by veterinarians. Although only clinical and radiographic data from the first postoperative week are reported here, ongoing monitoring, including behavioral observation, pain assessments, biomarker analyses, and weekly radiographic imaging, was performed throughout the protocol-defined study period to characterize the fracture model and ensure high standards of animal welfare.

### 2.2. Animals 

Eight skeletally mature, healthy, female Texel sheep (5.5 years ± 1 month, 76 kg ± 7 kg) were included (Table 1). The animals were housed indoors in 6 m^2^ pens, with a flat ground with wood shavings for bedding, and fed according to requirements. During the 4-week pre-surgery habituation period, health assessments were conducted using the European Animal Welfare Indicators (AWIN) assessment protocol for sheep [12]. Routine blood and fecal analyses were performed; the animals were treated with Albendazole upon indication (4 mg/kg BW, Valbezen Vet., Zoetis Animal Health ApS, Copenhagen, Denmark) before surgery.

### 2.3. Surgical Procedure and Post-Surgery Care

Following 24-36 h fasting and 4 h of water deprivation, the sheep were sedated with xylazine (0.12 mg/kg BW, IV, Xysol Vet., ScanVet Animal Health A/S, Fredensborg, Denmark) and butorphanol tartrate (0.1 mg/kg BW, IV, Dolorex, MSD Animal Health A/S, Copenhagen, Denmark). Anesthesia was induced with ketamine hydrochloride (2.5 mg/kg BW, IV, Ketador Vet., Salfarm Danmark A/S, Kolding, Denmark) and midazolam hydrochloride (0.1 mg/kg BW, IV, Midazolam Hameln, Hameln Pharma GmbH, Hameln, Germany). Anesthesia was maintained with isoflurane (1.5%, Vetflurane, Virbac Danmark A/S, Kolding, Denmark). Antibiotics were administered perioperatively: gentamicin sulfate (6.6 mg/kg BW, IV, Gentavet, ScanVet Animal Health A/S, Denmark) and benzylpenicillin natrium (22,500 mills. IU/kg BW, IV, Benzylpenicillin, Panpharma Nordic AS, Thisted, Denmark). Tetanus antitoxin (1500 units/animal, SC, Tetanus antitoxin Equine Origin, Colorado Serum Company, Denver, CO, USA) was administered before surgery. Analgesia consisted of flunixin meglumine (2.2 mg/kg BW, IV, Wellicox, Ceva Animal Health A/S, Vejle, Denmark) and a nerve block on each front limb with bupivacaine hydrochloride (0.1 mg/kg BW, SC, Bupivacaine Baxter, Baxter A/S, Søborg, Denmark) and mepivacaine hydrochloride (0.4 mg/kg BW, SC, Mepidor Vet, Salfarm Danmark A/S, Denmark).

**Figure 1:**
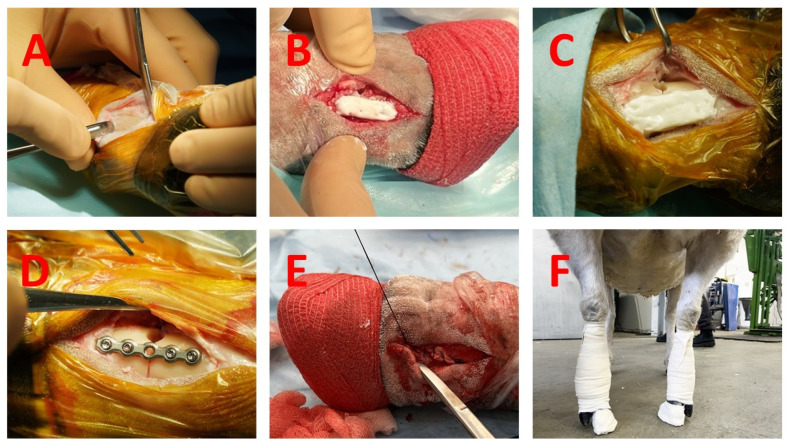
Surgical procedure of the composite patch technique (CPT) and the locking plate (LP): (**A**) skin and soft-tissue incision and bone exposure, (**B**) CPT ‘2 by 2’ configuration before skin closure, (**C**) CPT ‘3 by 3’ configuration before skin closure, (**D**) LP positioning before skin closure, (**E**) soft-tissue envelope closed before skin closure, (**F**) off-loading wooden block under each medial (non-operated) toe.

The sheep were positioned in dorsal recumbency with both front legs flexed and stabilized. A tourniquet was applied to the mid-metacarpal area of each limb. A dorsolateral skin incision was made, and the bone was exposed via blunt dissection and periosteum elevation. The extensor tendons were retracted, and the flexor tendons were protected with a curved retractor (Figure 1A). A 4.5 mm drill was used to create a dorsal mid-diaphyseal unicortical defect, followed by a complete transverse osteotomy with a 0.6 mm thick, 6 mm wide conical saw blade (DePuy Synthes, Switzerland). The circular defect was introduced to enable characterization of the fracture model in a concurrent study. The LP or CPT was then applied (Figure 1B–D). The soft tissue envelope and skin were closed with simple continuous (Novosyn 2/0, B Braun Medical Inc., Bethlehem, PA, USA) and simple interrupted sutures (Optilene 2/0, B Braun Medical Inc., USA) (Figure 1E). The same team of orthopedic and veterinary surgeons performed all procedures (TC, PH and SJ). A half-limb walking cast was applied in animals A1–A4. A wooden block adjusted to fit an ovine digit, 20 mm in height (Hoof shoe, standard, 26 mm height, Klovshoppen, Lem, Denmark), was glued under the medial digit on both front limbs for off-loading of the osteosynthesis in animals B1–B4. Additionally, an elastic bandage was applied for added support (Figure 1F).

After surgery, the sheep received gentamicin sulfate once daily (6.6 mg/kg BW, IV, Gentavet, ScanVet Animal Health A/S, Denmark) and benzylpenicillin natrium every 6 h (22,500 mills. IU/kg BW, IV, Benzylpenicillin, Panpharma Nordic AS, Denmark) for three days, and flunixin meglumine (2.2 mg/kg BW, IV, Wellicox, Ceva Animal Health A/S, Denmark) twice daily the first three days and once daily until day eight. Supplementary analgesics (additional doses of flunixin meglumine, butorphanol tartrate, and nerve block with bupivacaine applied at the mid-metacarpal level) were provided as needed. Euthanasia was performed by IV pentobarbital (140 mg/kg BW, Euthasol vet., Dechra Veterinary Products A/S, Uldum, Denmark). Sheep A4 was euthanized six days after surgery due to systemic illness.

### 2.4. Implants

The CPT involves the use of a prototype composite made of specialized trifunctional allyl and thiol triazine-trione monomers, a photo-initiator, and hydroxyapatite (Biomedical Bonding AB, Stockholm, Sweden). It is solidified using a handheld high-energy visible light lamp (Bluephase Powercure, Ivoclar Vivadent, Schaan, Liechtenstein) and affixed to the bone using unicortical titanium cortex screws (1.5 mm Medifit Devices LLP, Ahmedabad, India). The composite has demonstrated a modulus of elasticity of 6.6 ± 0.2 GPa and a tensile strength of 69 ± 3 MPa in initial mechanical testing [6,7]. The chemical composition and preparation protocol of the CPT material used in this in vivo study were identical to those used in previously published ex vivo studies. However, a complete mechanical characterization of the specific production series of materials used in this study was not conducted prior to the execution of this protocol; we therefore refer to previously published manuscripts for details regarding material composition and intrinsic mechanical behavior [6,7].

**Figure 2:**
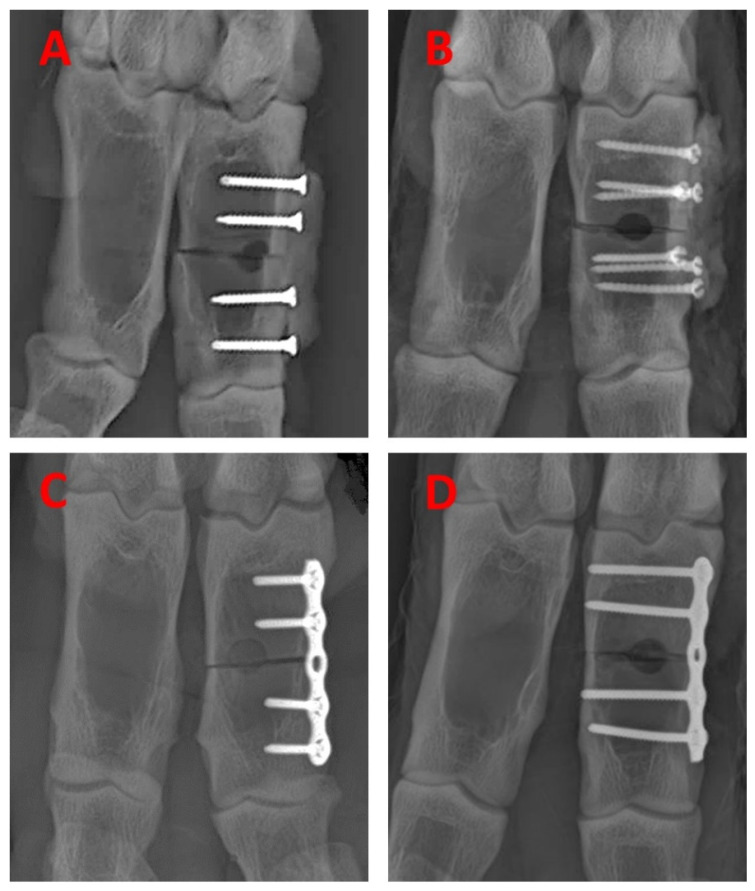
Intra-operative X-ray images illustrating the fracture model (diaphyseal osteotomy with 4.5 mm central defect) and the implant configurations employed: (**A**) Composite patch technique (CPT) ‘2 by 2’ configuration, (**B**) CPT ‘3 by 3’ configuration, (**C**) Locking plate (LP) with unicortical locking head screws, and (**D**) LPC with bicortical locking head screws.

The CPT was applied in two configurations: a ‘2 by 2’ with two screws per fragment, spaced 5 mm apart and 5 mm from the fracture line (sheep A1–A4) (Figure 2A), or a ‘3 by 3’ with three screws in a triangular arrangement, 5 mm apart and 5 mm from the fracture line (sheep B1–B4) (Figure 2B) [6]. Three layers of the composite were applied and cured incrementally to obtain a rectangular patch measuring approximately 25 mm × 8–10 mm. The LP was affixed with two locking screws in each fragment: unicortically (sheep A1–A4), (Figure 2C) or bicortically (sheep B1–B4), (Figure 2D).

### 2.5. Imaging 

X-rays were obtained in three projections: dorsopalmar, dorsomedial-palmerolateral oblique, and lateromedial (Gierth TR80/20: 60 kV, 3 mass, Fujifilm FDR D-EVO consol advanced software v.9) with the sheep sedated with xylazine (0.08 mg/kg BW, IV, Xysol Vet., ScanVet Animal Health A/S, Denmark) and butorphanol tartrate (0.04 mg/kg BW, IV, Dolorex, MSD Animal Health A/S, Denmark).

**Figure 3:**
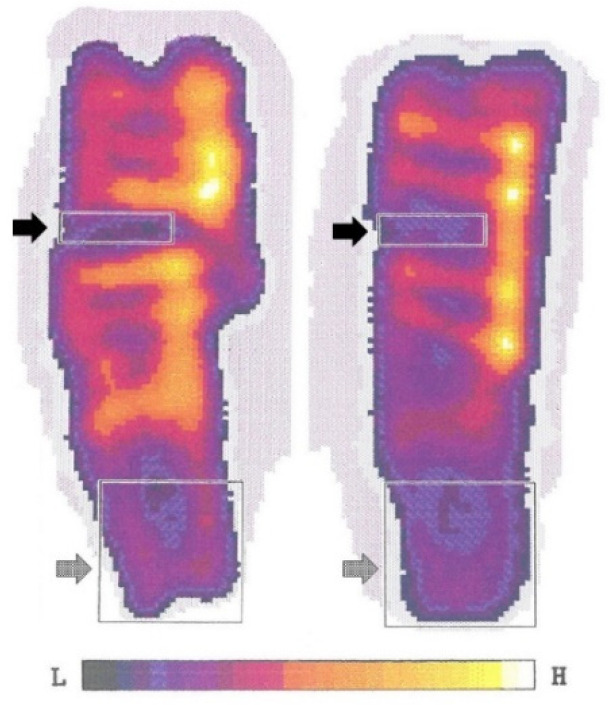
Example of post-euthanasia bone mineral density (BMD) analysis of the proximal phalangeal bone osteosynthesis with the composite patch technique (CPT), to the left, and locking plate (LP), to the right, respectively. The black arrow marks the fracture, and the grey arrow marks the distal epiphysis.

DXA scans (scan speed: 60 mm/s and a pixel size: 0.5 × 0.5 mm) were obtained using a Norland XR-46 bone densitometer (Norland Corp, Fort Atkinson, WI, USA) after euthanasia to assess BMD in the fracture area and distal epiphysis. BMD (g/cm^2^) was calculated by analysing the bone mineral content (g) of identical rectangular areas across all the samples: 0.42 cm^2^ in the fracture area and 1.5 cm^2^ in the distal epiphysis (Figure 3). Before scanning, each phalanx was disarticulated, the skin was removed, the soft tissue retained, and placed in 70% ethanol for 14 days.

### 2.6. Statistics

Descriptive statistics, mean and range, were calculated for BMD. The paired Wilcoxon Signed Rank Exact Test was applied to compare BMD across the two main treatment groups: the CPT and LP. Statistical significance was defined as *p* < 0.05. Statistical analyses were conducted using RStudio version 4.3.0 (RStudio Team, 2024).

## 3. Results

### 3.1. Radiological Evaluation 

Intraoperative radiographs confirmed satisfactory fracture reduction and implant placement, defined by proper alignment, angulation, rotation, and adequate apposition with a minimal gap (Figure 2A–D).

**Figure 4:**
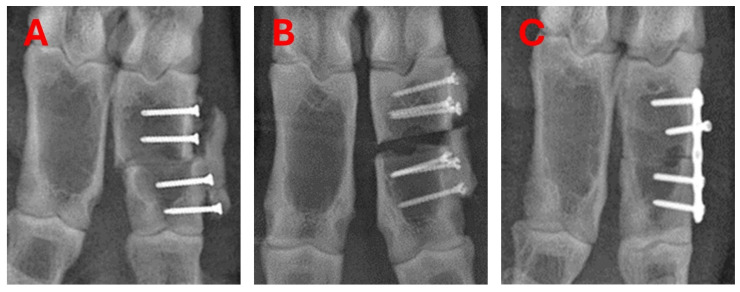
Examples of failure modes recorded at the 1 week mark. (**A**) composite patch technique (CPT) (sheep A1) showing patch breakage around screws. X-ray is taken through a cast, (**B**) CPT (sheep B1) showing patch breakage at midline. X-ray taken through an elastic bandage, and (**C**) Locking plate (LP) (sheep A3) showing the one example of screw loosening. X-ray taken through a cast.

In all sheep (n = 8), X-rays obtained 1 week after surgery demonstrated failure of the CPT by patch breakage; midline (n = 6), at the screw heads (n = 1); or both failure modes simultaneously (n = 1) (Table 1 and Figure 4A–B). No differences were observed between the ‘2 by 2’ and ‘3 by 3’ CPT configurations. At 1 week, all LP implants remained intact except for the loosening of a single screw (sheep A3) (Table 1 and Figure 4C). Despite the failure of the CPT, the sheep were maintained for the duration of the originally planned study period (16 weeks) to characterize the novel fracture model (e.g., healing of the LP repair and the multimodal animal welfare assessment). Lameness scores remained low despite the failure of the CPT. These results are beyond the scope of this paper and will be presented elsewhere.

### 3.2. DXA Analysis

**Figure 5:**
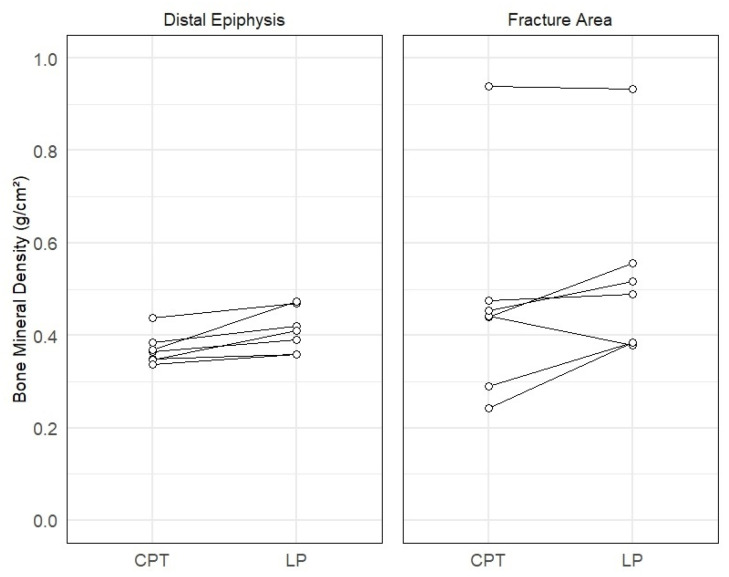
Paired comparison of bone mineral density (BMD) in seven sheep treated with the composite patch technique (CPT) or conventional locking plate (LP). BMD was measured at two locations: the fracture area and the distal epiphysis. Each line represents a paired measurement from the same animal. Note that values are presented on a unified *y*-axis.

Mean BMD values in the fracture area were 0.45 g/cm^2^ (range: 0.25–0.88) and 0.60 g/cm^2^ (range: 0.28–0.93) in the CPT and LP groups, respectively (*p* = 0.078). Mean BMD values in the distal epiphysis of each phalanx were 0.37 g/cm^2^ (range: 0.34–0.42) for CPT and 0.41 g/cm^2^ (0.36–0.48) for the LP (*p* = 0.016), Figure 5.

### 3.3. Animal Health and Welfare

After surgery, six sheep demonstrated normal demeanour, appetite, fecal output, and ruminal motility. Two sheep developed diarrhea and depression; one recovered by day 4 after IV isotonic fluid treatment (sheep A3). The other was euthanized on day 6 (sheep A4). Body temperatures within reference values and heart rates between 44 and 124 beats per minute were recorded. CSS remained below 3 (out of 10) for the six sheep, with scorings of 0 or 1 in four sheep. The two systemically affected sheep had CSS scores of 5–6 on day 1. These declined, and from day 6 onwards, all surviving sheep had generally low CSS scores. Lameness scores were below 1 (out of a maximum of 4) for all but two sheep, which scored 2–3 on two days.

## 4. Discussion

This study represents the first large-animal in vivo evaluation comparing the stability of the CPT to the LP in a novel experimental ovine phalanx fracture model. All CPT implants had failed at week 1, while the LPs were intact, except for one case of screw loosening. The study was continued until protocol-planned termination at week 16 to characterize the novel fracture model, with the animals under close observation and with adherence to conduct of safe animal welfare for the entire period. During the study, we added ad hoc modifications to the study design increasing stability to the CPT by changing the type of coaptation and screw configuration in the CPT and LP, as an attempt to decrease the risk of implant failure.

Previous studies demonstrated comparable biomechanical performance of the CPT and the LP in certain loading modalities, indicating that both osteosyntheses were likely to remain stable or were adequately stable to maintain reduction in vivo, thus justifying testing in an in vivo model. To understand the cause of the failures, the complex forces acting on the fracture site in vivo, as well as the mechanical and structural properties of the CPT, must be considered.

While ex vivo biomechanical evaluations provide initial insights into the mechanical performance of the CPT, they are limited to controlled loading conditions. In vivo, bones and implants are subjected to complex and dynamic forces, including muscle contractions, limb movements, shear, and compression. This may introduce additional biomechanical challenges for the CPT that are not accounted for in static ex vivo tests [13,14,15,16]. Failure of the CPT could be caused by incidental loads beyond the previously reported biomechanical ultimate threshold or by other uncontrolled forces of the extremity in this fracture model, despite protection with the cast or wooden hoof shoe. However, since only a single screw loosening was observed without further mechanical failure in the LP group, the biomechanical threshold was exceeded only in the CPT group.

While the previously demonstrated high stiffness of the CPT in reduced fractures can provide initial stability, it is coupled with low ductility compared to the LP [7]. Ductile materials can undergo significant plastic deformation before failure by effectively absorbing and redistributing stress, managing stress beyond the yield point, and reducing the risk of failure [17,18]. In contrast, less ductile materials like the CPT are more prone to stress concentrations and the propagation of micro-cracks under loading above the yield point, leading to failure [7,16]. Accordingly, this mechanical characteristic of the CPT likely contributed to the failure of this study.

The biomechanical performance of the CPT is influenced by its structural properties, including patch size, shape, thickness, and position and number of screws [6,7,19]. We attempted to increase biomechanical stability ad hoc by changing to the ‘3 by 3’ configuration, though the CPT could not be further enlarged without compromising soft-tissue closure in this model. This size limitation is particularly relevant in fractures where important structures, such as nerves and tendons, are in proximity or when soft-tissue coverage is minimal [20]. Despite these efforts, the CPT failed, indicating that the biomechanical capacity was exceeded in this model. Assessment of other CPT configurations is relevant in future studies, as it may improve biomechanical performance. Another consideration in this context is the customizability of the CPT. Though it allows for a custom fit, it can lead to variability in biomechanical stability, and such variations might have contributed to the observed failures [19]. To minimize this variation and for methodological consistency, the same team of surgeons performed all the procedures.

Exposure to cyclic loading must also be considered. Although the CPT previously demonstrated stability comparable to the LP under low to moderate cyclic loads, the failure in this study might be due to higher cyclic loads despite the protective measures [6]. In this fracture model, unrestricted movement after surgery without a sling was allowed. While this minimizes restraint, it introduces variability in fracture loading. In human patients, compliance with restrictions after surgery is generally expected to protect the fixation during early healing. However, certain patient populations, such as pediatric patients and individuals with dementia, psychiatric disorders, or alcohol dependence, may not fully adhere to such restrictions. This may lead to uncontrolled loading, increasing the risk of failure of the CPT [21,22,23].

The failures of the CPT implants were all recorded on the 1 week X-ray and further indicated by the post-euthanasia DXA analysis. The BMD levels in the CPT group were lower within the fracture area and statistically significantly lower in the distal epiphysis compared to the LP group. This underlines that the LP, despite screw loosening, maintained sufficient fracture reduction and indicates that these phalanges were subjected to higher loads in the follow-up period, implying reduced pain and fewer loading restrictions [24].

Despite the failure of the CPT in this study, certain features of the technique may still offer advantages in specific clinical scenarios. Its in situ customizability allows adaptation to complex fracture geometries or anatomies where conventional pre-formed implants may not achieve adequate fit [4,25,26]. Moreover, if mechanical loads in a given fracture remain below the material’s yield point, the CPT may provide sufficient fixation stability while permitting an appropriate degree of interfragmentary strain, as indicated in a previous in vivo rodent femur fracture model that healed with callus formation [6].

This study is not without limitations. As an exploratory investigation, this study provides early-stage insight, but further preclinical studies are required to confirm the finding. The sample size in this study is limited, but as the animals served as their own control, we considered a sample of 8 sheep (16 samples) to be in line with that of other animal studies in the field [11,27,28]. A priori power calculation was not performed, and these exploratory results should be interpreted cautiously, particularly given early implant failure in the CPT group. Despite protective measures applied in the fracture model, variable in vivo loading likely occurred, which may have influenced outcomes. Additionally, heterogeneity in implant configurations (e.g., CPT configuration and LP cortical engagement) and coaptation strategies was introduced during the study in an effort to improve CPT performance, but this complicates direct comparisons and may introduce bias. Finally, while a fracture model for low-load-bearing tubular bones is lacking, this study represents a first step in addressing this gap and offers early insight into the clinical limitations of the tested CPT.

## 5. Conclusions

In this exploratory study, the CPT demonstrated indications of inferior biomechanical stability compared to the LP in this fracture model, suggesting limited clinical applicability for fractures in human-weight-bearing or higher-load-bearing bones. Furthermore, the CPT should be used with caution in fractures that may be exposed to unpredictable loading, such as in pediatric or non-compliant patients. The one case of screw loosening in the LP group indicates that unforeseen incidental forces were acting on the fracture site in this model. Accordingly, the CPT might provide sufficient biomechanical stability to treat low-load-bearing fractures that are not exposed to such unpredictable loads or as an augmentation to other osteosynthesis techniques. Safe conclusions regarding clinical use cannot be drawn from the findings of this study, and further preclinical ex vivo evaluations are warranted before clinical applications of the CPT.

## Figures and Tables

**Table 1:** Overview of study design, including implant configurations for the locking plate (LP) and composite patch technique (CPT), description of implant failure modes at the 1 week mark and post-surgical coaptation (aftercare).

Sheep	Implant Configuration		1-Week Failure Mode		Aftercare	
	CPT	LP	CPT	LP	Wooden block	Cast or bandage
A1	2 by 2	Unicortical	Patch breakage (screw head)	No failure	Week 6–10	Cast, week 0–6. Bandage, week 6–8.
A2	2 by 2	Unicortical	Patch breakage (midline)	No failure	Week 3–10	Cast, week 0–3. Bandage, week 3–8.
A3	2 by 2	Unicortical	Patch breakage (midline)	Screw loosening	Week 4–16	Cast, week 0–4. Bandage, week 4–8.
A4	2 by 2	Unicortical	Patch breakage (midline)	No failure	*	Cast, week 0–1
B1	3 by 3	Bicortical	Patch breakage (midline)	No failure	Week 0–10	Bandage, week 0–8
B2	3 by 3	Bicortical	Patch breakage (midline)	No failure	Week 0–10	Bandage, week 0–8
B3	3 by 3	Bicortical	Patch breakage (midline and at screw heads)	No failure	Week 0–10	Bandage, week 0–8
B4	3 by 3	Bicortical	Patch breakage (midline)	No failure	Week 0–10	Bandage, week 0–8

* Euthanasia after 6 days due to post-surgical systemic illness.

## Data Availability

The original data presented in the study are openly available in Zenodo at https://doi.org/10.5281/zenodo.15431876.
